# Characterization of *Clostridium tyrobutyricum* Strains Using Three Different Typing Techniques

**DOI:** 10.3390/microorganisms8071057

**Published:** 2020-07-16

**Authors:** Johanna Burtscher, Franziska Küller, Matthias Dreier, Emmanuelle Arias-Roth, David Drissner, Konrad J. Domig

**Affiliations:** 1 Institute of Food Science, University of Natural Resources and Life Sciences Vienna (BOKU), 1190 Vienna, Austria; franziska@kueller.eu (F.K.); konrad.domig@boku.ac.at (K.J.D.); 2 Competence Centre for Feed and Food Quality, Safety and Innovation (FFoQSI), Competence Centre for Feed and Food Quality, Safety and Innovation, 3440 Tulln, Austria; 3 Agroscope, 3003 Bern, Switzerland; matthias.dreier@agroscope.admin.ch (M.D.); emmanuelle.arias@agroscope.admin.ch (E.A.-R.); drissner@hs-albsig.de (D.D.); 4 Department of Life Sciences, Albstadt-Sigmaringen University, 72488 Sigmaringen, Germany

**Keywords:** *Clostridium tyrobutyricum*, cheese, spoilage, typing, fingerprinting, MALDI-TOF MS

## Abstract

*Clostridium tyrobutyricum* is well known as one of the main causative agents of severe cheese spoilage. The metabolism of this anaerobic bacterium during ripening leads to textural and sensory defects in cheese and consequential loss of product value. The potential to induce cheese spoilage, however, may vary among different strains of the same species. Therefore, a better understanding of the intra-species diversity of *C. tyrobutyricum* may be of practical relevance for the dairy industry. In the present study, we compared the ability of three typing techniques to differentiate 95 *C.* *tyrobutyricum* strains on the subspecies level: (1) repetitive element palindromic PCR (rep-PCR) fingerprinting combined with conventional agarose gel electrophoresis, (2) hexaplex-PCR followed by an automated capillary electrophoresis and (3) matrix-assisted laser desorption ionization-time of flight mass spectrometry (MALDI-TOF MS) typing. MALDI-TOF MS fingerprinting provided only moderate reproducibility and low discriminatory power. Both PCR-based methods were highly reproducible and discriminative, with hexaplex-PCR fingerprinting being slightly more discriminative than rep-PCR typing. Overall, a high intra-species diversity was observed among the tested strains, indicating that further investigations on the strain level may be of interest.

## 1. Introduction

*Clostridium tyrobutyricum* is recognized as one of the main causative agents of severe cheese spoilage. Endospores of this anaerobic bacterial species enter the raw milk during the milking process and may germinate and outgrow under favorable conditions during cheese ripening. The principal products of clostridial metabolic activity in cheese are gases (H_2_ and CO_2_) and organic acids (mainly butyric acid) [1]. The presence of these metabolites leads to rancid off-flavors and pronounced textural defects in cheese, such as slits, cracks and undesired eyes [2,3]. Due to these severe quality defects, which are summarized under the term ‘late blowing’, cheese producers may face a considerable loss of product value and revenue.

Many studies have investigated the clostridial diversity in the dairy environment to gain a better understanding of how clostridia may affect cheese quality. In this context, naturally occurring clostridial populations along the dairy supply chain have been characterized in depth on the species level [4,5,6,7,8,9,10,11,12,13,14,15,16,17,18,19]. One of the main conclusions of the listed studies was that the species *C. tyrobutyricum* is particularly relevant for cheese spoilage. However, although some of the techniques used in the studies listed above would be suitable for bacterial fingerprinting (e.g., randomly amplified polymorphic DNA (RAPD)-PCR, automated ribosomal intergenic spacer analysis (ARISA) or amplified 16S ribosomal DNA restriction analysis (16S ARDRA)), only a few studies have devoted special attention to the differentiation of butyric acid-producing clostridia on the subspecies or strain level [1].

For a long time, the application of pulsed field gel electrophoresis (PFGE) has been considered as the ‘gold standard’ for bacterial strain typing, but this method turned out to be challenging for clostridia due to a high occurrence of endogenous bacterial nucleases and an incomplete lysis of bacterial cells [20,21]. Garde et al. developed an improved PFGE protocol and not only found a high genomic diversity among isolated clostridial species from Manchego cheese, but also among isolates belonging to the same species [22]. Nevertheless, despite its high discriminatory power and high reproducibility, PFGE is not used preferably for large sample sets due to its labor intensity and complexity [21]. A more recent technique, multilocus variable-number of tandem repeat (VNTR) analysis, exploits the fact that the number of tandemly repeated DNA sequences per locus may vary strongly among strains within a given species [21]. Nishihara et al. developed a multiple loci variable-number of tandem repeat analysis (MLVA) protocol with the aim to use this typing technique to understand the contamination pathways of *C. tyrobutyricum* along the dairy supply chain [23]. The authors observed a high diversity among the 25 tested *C. tyrobutyricum* isolates but, to the best of our knowledge, this MLVA method has not been applied in further studies. Among the well-known typing techniques, repetitive sequencing-based PCR fingerprinting is considered a very rapid and cost-effective approach [21]. Bermúdez et al. applied a repetitive element palindromic PCR (rep-PCR) approach using the primer (GTG)_5_ to investigate the population structure of 44 *C. tyrobutyricum* isolates and observed a high genetic diversity [24].

The described findings suggest that the intra-species diversity of the species *C. tyrobutyricum* merits further investigation. Matrix-assisted laser desorption ionization-time of flight mass spectrometry (MALDI-TOF MS) typing has emerged as a fast and simple tool for intra-specific typing. It has been applied successfully for the subtyping and tracking of several bacterial pathogens [25,26,27]. A more recent development is the use of MALDI-TOF MS to characterize food spoilage organisms [28]. In this context, it is important to consider that the discriminatory power of MALDI-TOF MS fingerprinting may vary according to the species studied, as some bacteria may be very heterogeneous on the subspecies level and others may be indistinguishable [29]. To the best of our knowledge, no data are available describing the performance of MALDI-TOF MS for the typing of butyric acid producing clostridia or *C. tyrobutyricum*.

We hypothesize that the spoilage potential of *C. tyrobutyricum* may be strain-dependent. Therefore, increased knowledge about the genetic diversity of *C. tyrobutyricum* may be not only of practical relevance for the dairy industry but also the basis for further scientific studies on cheese spoiling clostridia. Hence, the aim of the present study was to develop and evaluate simple and fast protocols that will be suitable for the investigation of a large number of *C. tyrobutyricum* strains on the subspecies level. For this purpose, three methods were established and compared: (1) (GTG)_5_-fingerprinting combined with conventional agarose gel electrophoresis, (2) a new MLVA approach using hexaplex PCR followed by an automated capillary electrophoresis and (3) MALDI-TOF MS typing.

## 2. Materials and Methods

### 2.1. Bacterial Strains and Growth Conditions

A total of 95 *C. tyrobutyricum* strains of the culture collection of the Institute of Food Science of the University of Natural Resources and Life Sciences Vienna were used for the comparison of the three typing methods. Besides the type strain DSM 2637 (named Cl_20 in this study), the strains DSM 663 (Cl_14), DSM 664 (Cl_15) and DSM 1460 (Cl_2) were obtained from the German Collection of Microorganisms and Cell Cultures. The strains NCDO 1759 (Cl_3) and NCIMB 701755 (Cl_55) had been received from the National Collection of Industrial, Food and Marine Bacteria in the UK. The strains Cl_25, Cl_29 and Cl_51 had been isolated from NIZO strain BZ 15 and three strains had been obtained from Agroscope in Switzerland in the 1970s, i.e., FAM1559 (Cl_33), FAM 25158 (Cl_52), and FAM25159 (Cl_53). The remaining 83 strains were isolated within our laboratory from 27 cheese samples from more than eight production locations (named A-H). The assignment of each strain to the species *C. tyrobutyricum* was based on 16S rDNA sequencing according to the procedure described by Brändle et al. [14]. The strains were stored at −80 °C in reinforced clostridial broth (RCM, Merck, Germany) supplemented with 20% (*v*/*v*) glycerol. For the analyses, the cultures were reactivated on reinforced clostridial agar (RCA, Merck, Germany) and incubated for 48 h at 37 °C under anaerobic conditions using a jar gassing system and a gas mixture containing 80% N_2_, 10% CO_2_ and 10% H_2_ (Don Whitley Scientific, West Yorkshire, UK).

### 2.2. MALDI-TOF MS Fingerprinting

Preliminary experiments showed that direct smearing and the extended direct transfer method for protein extraction often yielded low-quality spectra and low identification scores for *C. tyrobutyricum* (data not shown). Hence, for MALDI-TOF MS fingerprinting, bacterial proteins were extracted using the formic acid extraction procedure. One to five clostridial colonies were suspended in 300 μL of deionized water. Then, 900 µL of absolute ethanol were added and the suspension was thoroughly mixed. After a centrifugation step at 14,000× *g* for 2 min, the supernatant was discarded. Centrifugation was repeated as described above to remove residual ethanol and the supernatant was discarded again. The remaining pellet was dried at ambient temperature for 10 min and subsequently suspended in 5–20 µL 70% formic acid (volume depending on the pellet size). Then, the same amount of acetonitrile as formic acid was added and the mixture was thoroughly homogenized using a pipette. After a final centrifugation step at 14,000× *g* for 2 min, 1 µL of the supernatant was spotted in 3 replicates onto a steel target plate and left to dry at ambient temperature. Then, each sample spot was coated with 1 µL of HCCA matrix solution (2.5 mg of α-cyano-4-hydroxycinnamic acid in 250 µL of 50% acetonitrile with 2.5% trifluoracetic acid) and left to dry. On each target plate, the bacterial test standard (BTS) was included in one spot for calibration. On each analysis day, strain Cl_171 was included into the analysis as a control. Furthermore, the reproducibility of the method was tested by repeatedly reactivating a subset of 22 randomly chosen strains from the cryo cultures and comparing the obtained spectra from different measurements. The spectra were acquired using a Microflex LT mass spectrometer (Bruker Daltonics, Bremen, Germany) according to the standard settings recommended by the manufacturer (method ‘MBT_AutoX’). Each spot was measured three times. Hence, a total of 9 spectra were acquired for each strain. First, the obtained spectra were validated using the FlexAnalysis software (check of calibration constant, visual inspection of outliers or anomalies and peak shifts). Spectra that did not pass the quality control were deleted. Furthermore, correct assignment to the species *C. tyrobutyricum* was rechecked by comparing the spectra to the reference database. Subsequently, the remaining spectra were imported into the BioNumerics software v7.6.3. (Applied Maths, Ghent, Belgium) and pre-processed according to the default ‘strict preprocessing’ option. In the next step, summary spectra were created. In this step, technical replicates below a similarity threshold of 95% were inactivated for further analyses. The arithmetic mean of the number of remaining active technical replicates, that represented one summary spectrum, was 8.5. Peak matching was performed with a constant tolerance of 1.9, a linear tolerance of 550 and a peak detection rate of 10%. Clustering was performed based on the Pearson correlation and the unweighted paired-group method with arithmetic mean (UPGMA) method.

### 2.3. DNA Extraction

DNA from overnight cultures of *C. tyrobutyricum* strains in reinforced clostridial medium (Merck, Germany) was isolated using the peqGOLD bacterial DNA kit (VWR, Darmstadt, Germany).

### 2.4. Primer Design (Hexaplex PCR)

The draft genome data of five *C. tyrobutyricum* whole genome shotgun projects (ANOE01, BASR01, CBXI01, JTES01, JTER01) were downloaded from the National Center for Biotechnology Information (NCBI). The Tandem Repeats Finder program 4.07b (https://tandem.bu.edu/trf/trf.html) was used to search for VNTR loci in the draft genome sequences [30]. Flanking sequences of VNTR loci were used as templates for primer design with CLC genomics Workbench 8.0 (CLC bio, https://digitalinsights.qiagen.com). Primer pairs that showed amplification of discriminative VNTR loci in all draft genomes in-silico were selected. Primer pairs, primer concentrations and the annealing temperature for the hexaplex-PCR were then determined empirically. In vitro validation with 10 *C. tyrobutyricum* strains from the Agroscope Culture Collection showed discrimination of all strains.

### 2.5. Hexaplex-PCR Fingerprinting

In the PCR reaction, a mix of six primer pairs was used according to the description provided in Table 1. The PCR reaction consisted of a total volume of 25 µL including 12.5 µL AccuStart II PCR ToughMix (Qiagen, Hilden, Germany), 10.5 µL molecular grade water, 1 µL of the primer mix with the final concentrations described in Table 1 and 1 µL of DNA extracted from a pure culture of *C. tyrobutyricum*. The amplification was performed in a Mastercycler nexus SX1 (Eppendorf, Hamburg, Germany) as follows: denaturation at 94 °C for 10 min, followed by 35 cycles of denaturation at 94 °C for 30 s, annealing at 57 °C for 30 s and elongation at 72 °C for 30 s followed by a final elongation at 72 °C for 7 min. PCR products were analyzed using the Agilent DNA 1000 Kit in an automated electrophoresis system (2100 Bioanalyzer instrument, Agilent Technologies, Vienna, Austria). Gel images resulting from the electropherograms were imported into the Bionumerics software v7.6.3. Clustering of the band profiles was performed using Dice’s coefficient and the UPGMA method. An optimization of 0.6% and a tolerance level of 1% were specified to create dendrograms. The method’s reproducibility was assessed by amplifying DNA from 20 randomly chosen strains multiple times.

### 2.6. Rep-PCR Fingerprinting

A repetitive element palindromic PCR (rep-PCR) was performed using the primer pair (GTG)_5_. The PCR reaction mixture (total volume 25 µL) consisted of 2.5 µL of 10× PCR buffer (Tris-HCl (10 mM), KCl (150 mM), MgCl_2_ (1.5 mM), 0.1% Triton X-100; pH 8.8; Thermo Scientific), 0.5 µL deoxynucleotide triphosphate mix (0.2 mM each), 0.5 µL DynaZyme II DNA polymerase (2 U/µL) (Thermo Scientifc), 1 µL primer (GTG)_5_ (50 µM), 19.5 µL sterile distilled water and 1 µL of template DNA from pure cultures of *C. tyrobutyricum*. The amplification was performed in a Mastercycler ep (Eppendorf, Hamburg, Germany) with the following set-up: initial denaturation at 95 °C for 7 min, 30 cycles of denaturation at 90 °C for 30 s, annealing at 40 °C for 1 min and extension at 65 °C for 8 min, followed by a final extension at 65 °C for 16 min. PCR products were separated on an agarose gel (2%), stained using ethidium bromide, captured under UV light and imported into the BioNumerics software for further analyses. Clustering of the band profiles was performed using Dice’s coefficient and the UPGMA method. An optimization of 0.6% and a tolerance level of 1% were specified to create dendrograms. The reproducibility of the method was evaluated by amplifying DNA from 10 randomly chosen strains 2 to 3 times.

### 2.7. Statistics

The discriminatory power of each method was calculated using the Simpson’s index of diversity [31]. As a measure of agreement between the tested methods, Rand and adjusted Rand coefficients were calculated [32,33,34]. The Rand index measures the similarity between two clusterings and yields values between 0 and 1, whereas the adjusted Rand index assesses the overall congruence among typing methods adjusted for chance agreement and can have negative values [33,35].

## 3. Results

### 3.1. Method Reproducibility

**Figure 1:**
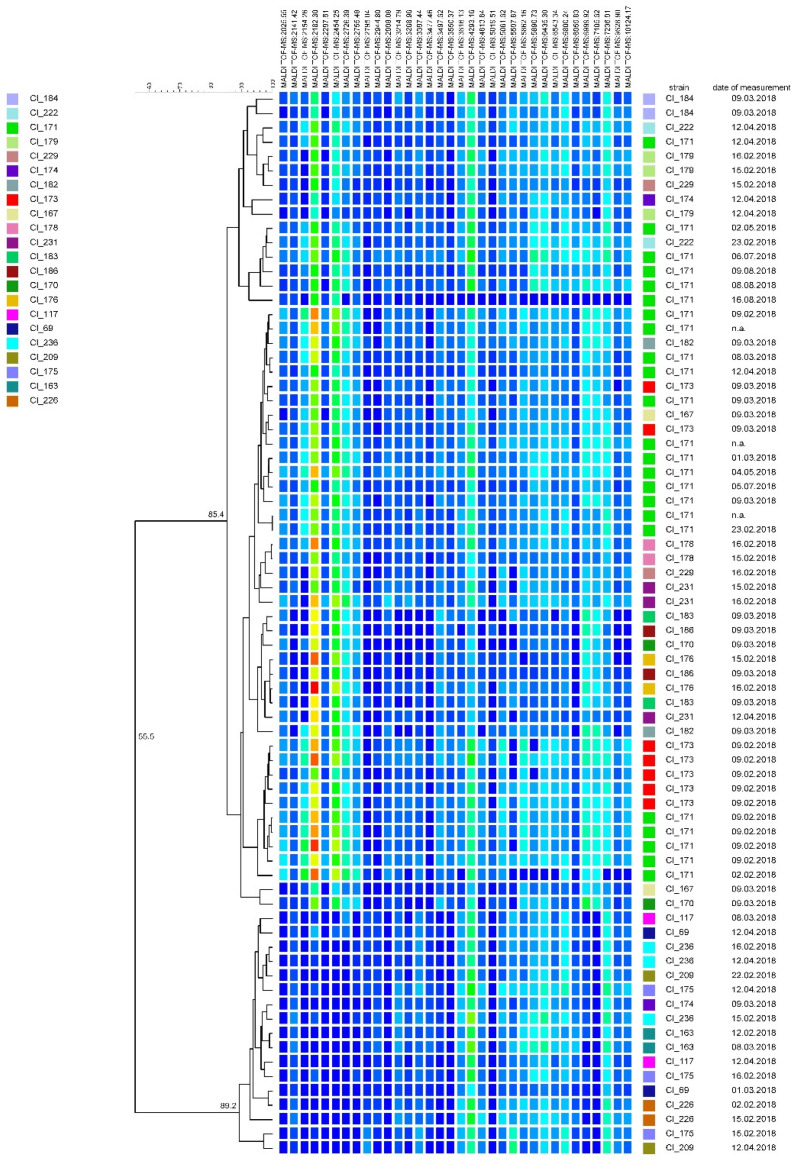
Dendrogram obtained from repeated matrix-assisted laser desorption ionization—time of flight mass spectrometry (MALDI-TOF MS) fingerprinting of 22 *C. tyrobutyricum* strains. The dendrogram was constructed using the unweighted pair group method with arithmetic mean (UPGMA) with similarity levels expressed as percentage values of the Pearson correlation coefficient. The color shading indicates peak intensity (from low to high intensity: dark blue, green, yellow, red); n.a.: not available.

Figure 1 depicts the results of repeated MALDI-TOF MS fingerprinting of 22 randomly selected strains. Although a curve-based clustering was performed, spectral data are provided in the form of a peak matching table due to the improved visibility of relevant peaks. Two major clusters can be differentiated from each other in this sample set. Each strain and its corresponding replicates, which have been summarized into a group and marked with a common color, are assigned to either cluster I or cluster II. Higher similarity levels can be observed among strains whose spectra were acquired on the same date. However, independently from the date of measurement, replicates of the same strain show a minimum similarity of 85%.

**Figure 2:**
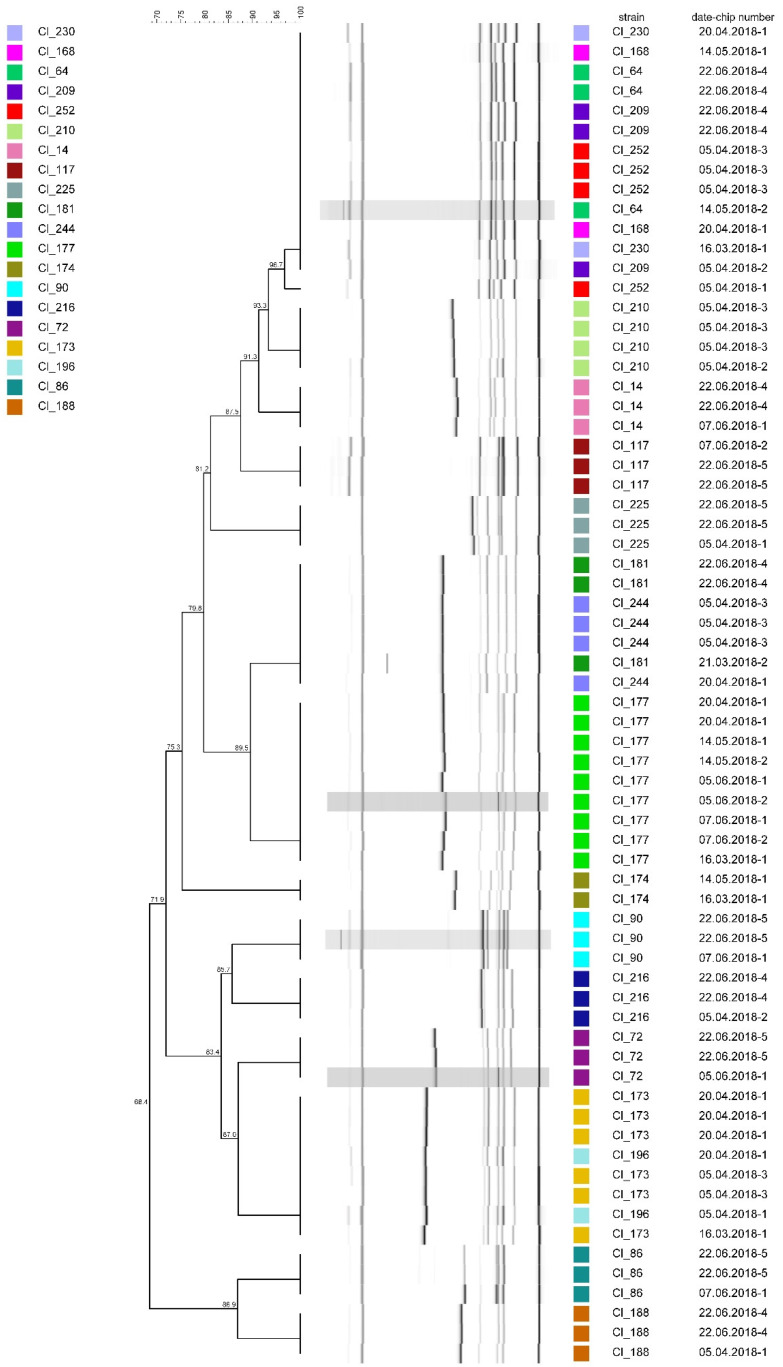
Dendrogram obtained from repeated hexaplex-PCR fingerprinting of 20 *C. tyrobutyricum* strains. The dendrogram was constructed using the unweighted pair group method with arithmetic mean (UPGMA) with similarity levels expressed as percentage values of Dice’s correlation coefficient.

In Figure 2, resulting band patterns from repeated analyses of 20 randomly selected strains using hexaplex-PCR typing are clustered according to their similarity. Replicates of the same strain are marked with the same color. Out of the 20 strains, 19 yielded band patterns of 100% similarity in all experiments regardless of the PCR run or the gel electrophoresis chip. Only one replicate of strain Cl_252 showed a lower similarity of 96.7% to a group of three other replicates, which had been tested simultaneously and yielded patterns of 100% similarity.

**Figure 3:**
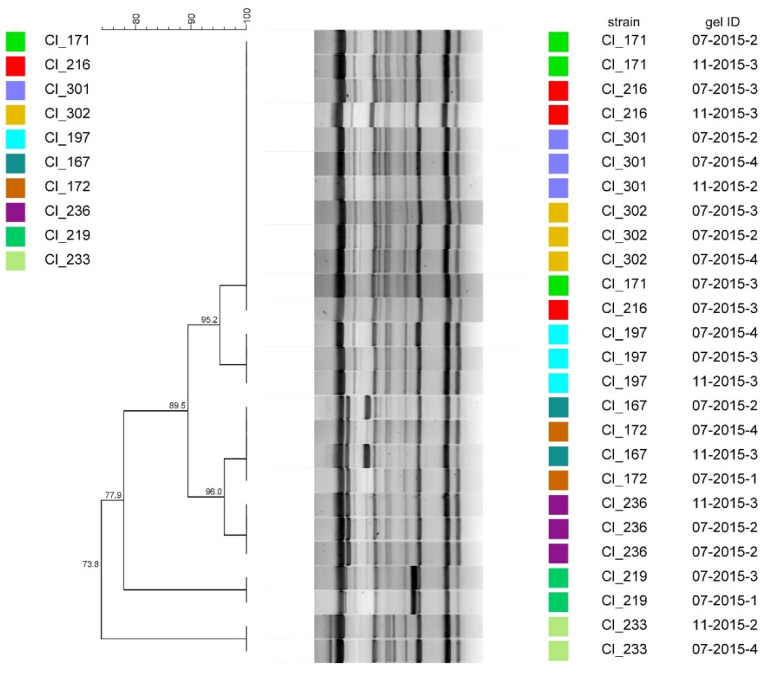
Dendrogram obtained from repeated repetitive element palindromic PCR (rep-PCR) (GTG)_5_ fingerprinting of 10 *C. tyrobutyricum* strains. The dendrogram was constructed using the unweighted pair group method with arithmetic mean (UPGMA) with similarity levels expressed as percentage values of Dice’s correlation coefficient. The column ‘gelID’ identifies the gel by the month and year of analysis and the gel number.

The results of the reproducibility testing of (GTG)_5_-fingerprinting by repeated analyses of 10 randomly selected strains are shown in Figure 3. Of each of the tested strains, all replicates showed a 100% concordance even though they had been amplified during different PCR runs and separated on distinct agarose gels.

### 3.2. Diversity of the Test Strain Set

**Figure 4:**
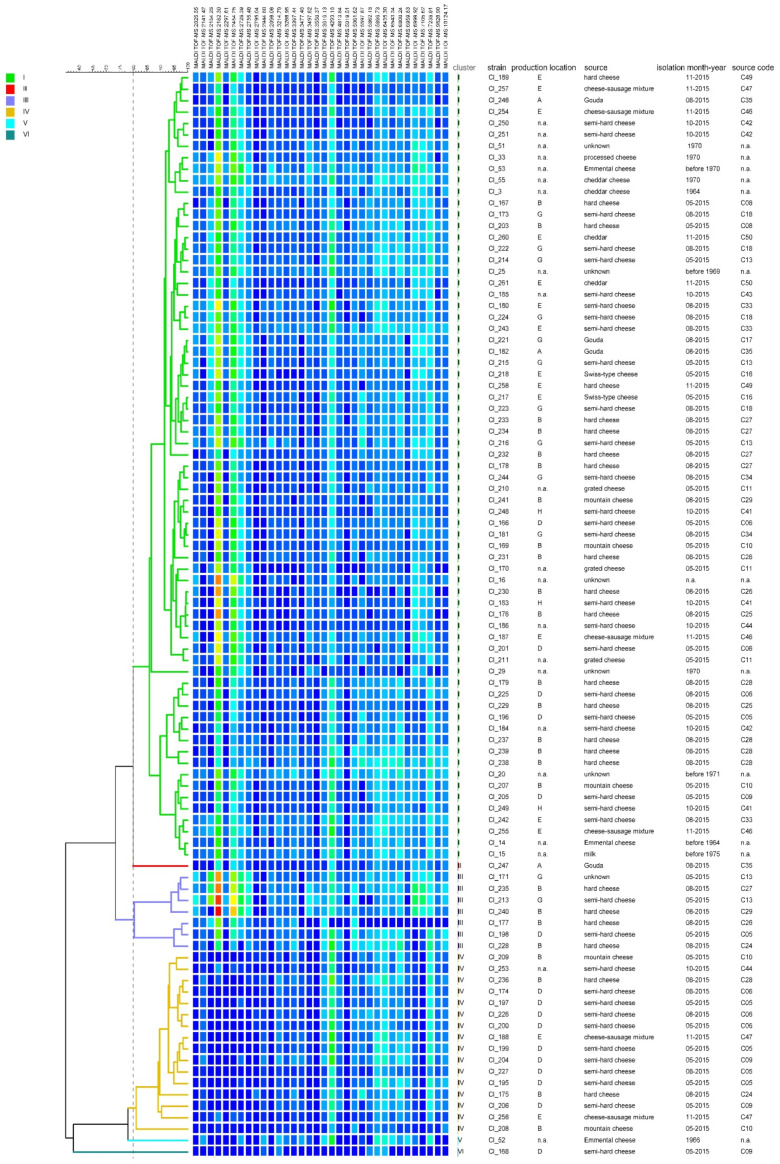
Dendrogram obtained based on the cluster analysis of the MALDI-TOF MS spectra of 95 *C. tyrobutyricum* strains. The dendrogram was constructed using the unweighted pair group method with arithmetic mean (UPGMA) with similarity levels expressed as percentage values of the Pearson correlation coefficient. The vertical grey dashed line indicates the threshold similarity level (80%) for cluster formation. The color shading indicates peak intensity (from low to high intensity: dark blue, green, yellow, red); n.a. not available.

Intra-species diversity among 95 *C. tyrobutyricum* strains was assessed using MALDI-TOF MS, hexaplex-PCR and rep-PCR fingerprinting. To visualize similarities, a cluster analysis was performed from the data sets obtained using each method. The dendrogram obtained from the cluster analysis of the MALDI-TOF MS spectra (Figure 4) is composed of six clusters referred to as cluster I to VI. Cluster I comprises most of the strains (N = 69), followed by clusters IV (N = 16) and III (N = 7). Clusters II, V and VI contain only one strain each. Isolates from the same production location or cheese sample (indicated by the source code) were distributed into different clusters. Simpson’s diversity index yielded 0.44, which indicates low discriminatory ability of MALDI-TOF MS fingerprinting considering that 0 means no diversity and 1 represents infinite diversity [31].

**Figure 5:**
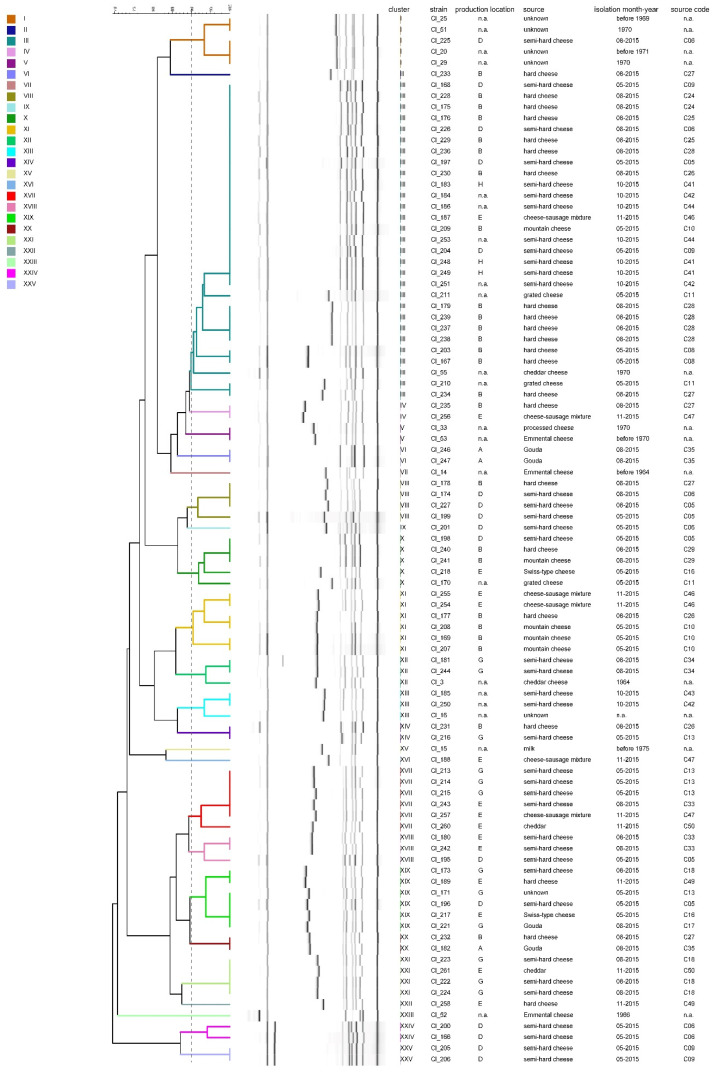
Dice clustering dendrogram of hexaplex-PCR analysis of 95 *C. tyrobutyricum* strains. The dendrogram was constructed using the unweighted pair group method with arithmetic mean (UPGMA) with similarity levels expressed as percentage values of Dice’s correlation coefficient. The vertical grey dashed line indicates the threshold similarity level (90%) for cluster formation. n.a.: not available.

Conversely, a higher diversity was observed among the fingerprints obtained using hexaplex-PCR (Figure 5). At a threshold of 90% similarity, the test strains grouped into 25 clusters, named I to XXV. Cluster II contains 30% of the strains (N = 29), whereas all other clusters consist of one to six strains. Several clusters (III, VI, VIII, X, XI, XII, XVII, XVIII, XXI, XXIV and XXV) contain, albeit not always exclusively, more than one isolate from the same cheese sample (indicated by the same source code). Interestingly, the fingerprints of most of the strains that had been isolated before 1970, including the type strain Cl_20 and strains Cl_14, Cl_25, Cl_51 and Cl_52, show low similarity with the fingerprints of more recently isolated strains. The significantly higher cluster number obtained using hexaplex-PCR compared to mass spectrometry data is also reflected in the significantly higher Simpson’s diversity index of 0.89.

**Figure 6:**
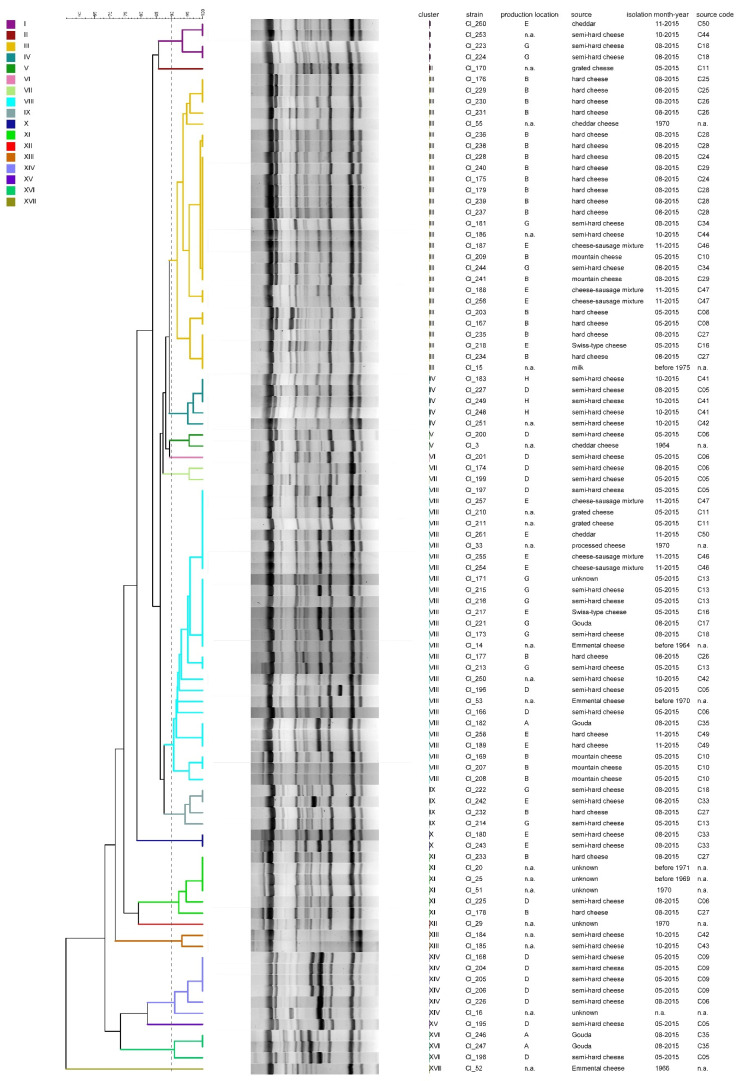
Dice clustering dendrogram of rep-PCR (GTG)5 fingerprints of 95 *C. tyrobutyricum* strains. The dendrogram was constructed using the unweighted pair group method using arithmetic mean (UPGMA) with similarity levels expressed as percentage values of Dice’s correlation coefficient. The vertical grey dashed line indicates the threshold similarity level (90%) for cluster formation. n.a.: not available.

The dendrogram obtained from rep-PCR results (Figure 6) is composed of 17 clusters in total, numbered from I to XVII. Two major clusters contain 27 strains each, whereas the other clusters form groups of one to six strains. Some clusters primarily consist of isolates from cheeses from the same production location (e.g., production location A in cluster XVI, B in cluster III, D in cluster VII and XIV, E in cluster X and H in cluster IV). However, it was not possible to define characteristic fingerprints specific for a production location or cheese sample. Strains isolated from the same cheese sample are often found within one cluster but are also distributed among different clusters. Rep-PCR analysis of the 95 strains yielded a diversity index of 0.83, which is slightly lower than the index obtained using hexaplex-PCR typing.

## 4. Discussion

### 4.1. Method Reproducibility

The guideline MM11-A of the Clinical and Laboratory Standard Institute (CLSI) states that the similarity coefficient of repeated measurements from molecular typing methods is ideally above 0.95, and replicates of the same strain should aggregate in the same cluster [36]. Hexaplex-PCR and rep-PCR fingerprinting both fulfilled these criteria and showed excellent reproducibility. However, it must be taken into consideration that bands from the automated gel electrophoresis were selected automatically using the BioNumerics software and only minor corrections were necessary. Conversely, for rep-PCR fingerprints, each band was selected manually and the selection was revised several times. Hence, the results of rep-PCR typing were strongly influenced by the operator who had prepared the gel and selected the bands on the fingerprints. The automated electrophoresis system used for the hexaplex-PCR typing attenuated the operator influence and facilitated subsequent bioinformatic analysis, but the expenses for the electrophoresis were significantly higher.

For MALDI-TOF MS fingerprinting, the definition of standard criteria, that apply for all bacteria, is complex and the discriminatory power of MALDI-TOF MS typing potentially has to be defined for each species [37]. Nevertheless, it is obvious that the repeated analyses of replicates of the same strains yielded lower similarities using MALDI-TOF MS fingerprinting than the PCR-based typing techniques. One explanation for the higher variability among replicates of the same strain using MALDI TOF MS could be that the proteome reflects the immediate environment. Hence, although culture conditions had been standardized, minor differences may have influenced the proteome, whereas the genome of a strain is considered to be more stable towards short-term influences during cultivation [29,38]. The findings from reproducibility tests were considered to define similarity thresholds for the subsequent diversity assessment performed in this study: an 80% similarity threshold was chosen for MALDI-TOF MS-based fingerprinting and a 90% similarity threshold for hexaplex and rep-PCR typing.

### 4.2. Diversity of the Test Strain Set

Comparing the data obtained using the three methods in this study, MALDI-TOF MS fingerprinting yielded the lowest diversity and hexaplex-PCR fingerprinting yielded the highest diversity among the tested strains. Some qualitative agreements can be observed among the three tested methods. For instance, the isolate Cl_52 is clustered separately from all the other isolates in the dendrogram of each method. Concordance became apparent particularly between the two PCR-based typing methods, as strains are clustered into similar groups and often the same groups of strains are indistinguishable from each other. For a quantitative evaluation of concordance between the three assessed fingerprinting techniques, Rand (RI) and adjusted Rand (ARI) indices were calculated. While the congruence between MALDI-TOF MS and hexaplex-PCR typing (RI 0.45, ARI 0.00) and MALDI-TOF MS and rep-PCR typing (RI 0.48, ARI 0.03) was very poor, hexaplex-PCR and rep-PCR fingerprinting yielded the greatest congruence (RI 0.8, ARI 0.2).

The low diversity of the test strain set, which has been observed using MALDI-TOF MS typing, is consistent with results from other studies. In the presented study, two clusters contained 90% of the strains from the test sample set. In a study performed by Illikoud et al. on 161 *Brochothrix thermospacta* isolates, one major cluster encompassed more than 70% of the analyzed strains [28]. MALDI-TOF MS typing is a fast and cost-effective alternative to other typing methods [39,40]. To the best of our knowledge, this is the first study that investigated intra-species diversity among cheese spoiling clostrida using this technique. Hovewer, the results show that the reproducibility of MALDI-TOF MS fingerprinting is low in comparison to the DNA- based methods or PFGE typing [22]. Ghyselink et al. observed a high discriminatory power of MALDI-TOF MS but also reported that rep-PCR yielded better strain differentiation than MALDI-TOF MS when members of the genera *Rhizobium*, *Streptomyces*, *Paenibacillus*, *Arthrobacter* and *Pseudomonas* were analyzed [29,41]. However, it is important consider that the results of this study do not indicate that a strain-level resolution generally cannot be obtained by applying MALDI-TOF MS. Alternative sample preparation methods (e.g., using enzymes or mechanical force), other mass spectrometry technologies or bioinformatics tools may yield a higher resolution and enable better strain differentiation [27,29,42,43].

Within the present study, rep-PCR typing and hexaplex-PCR typing were highly reproducible and both methods yielded high intra-species diversity. The highest discriminatory coefficient was obtained using hexaplex-PCR typing (0.89). This value is still lower than the diversity indices obtained from PFGE (0.92–0.95) or MLVA (0.96) typing in other studies [22,23,44]. However, in this respect, we want to point out that comparisons of diversity coefficients obtained from different sample sets have to be interpreted with caution [23]. Despite the superior discriminatory ability of PFGE or MLVA typing in other studies, the hexaplex-PCR typing method presented in this study stands out as an interesting fast and simple alternative to PFGE but also to rep-PCR typing.

None of the three evaluated methods in this study enabled the identification of source-specific fingerprints within the selected test sample set. Similarities were observed among isolates from the same environment, but most of the clusters, which encompassed more than two strains, consisted of a mixture of isolates from different sources. Thus, the tested methods are of limited use for tracking contamination pathways. However, a screening of isolates using these fast and simple procedures may provide interesting insights into the diversity of *C. tyrobutyricum* strains from different origins and may serve as a basis for further genomic or proteomic investigations.

## 5. Conclusions

The aim of this study was to establish a simple protocol for the differentiation of a large number of *C. tyrobutyricum* strains on the subspecies level. Two new approaches, namely MALDI-TOF MS fingerprinting and hexaplex-PCR fingerprinting, as well as conventional rep-PCR typing, have been evaluated. Despite being simple and cost-effective, MALDI-TOF MS fingerprinting provided only moderate reproducibility and low discriminatory power. Both PCR-based methods were highly reproducible and discriminative, with hexaplex-PCR fingerprinting being slightly more discriminative than rep-PCR typing. In agreement with other studies, high intra-species diversity among the tested *C. tyrobutyricum* strains was observed. Whether and how the observed differences among the strains affect phenotypic characteristics requires further research. In the long term, increased knowledge about the genotypic and phenotypic traits of *C. tyrobutyricum* will improve our understanding of this species’ role as a causative agent of major cheese spoilage.

## Figures and Tables

**Table 1:** Primers used in this study for the hexaplex-PCR analysis.

Primer Name	Primer Sequence (5′ → 3′)	Concentration [µM]
CTM1 F	ATTACTCAAGCCGCCAAT	0.08
CTM1 R	CGGGTCATGGAATACTGAA	0.08
CTM5 F	GGAAAAGAACTTCCAGGAAT	0.25
CTM5 R	CTGTATCCGAAAATCCTCATTA	0.25
CTM6 F	CGACAACAGCGATATAACAAA	0.10
CTM6 R	GCCTTTTCACCATTTCCAT	0.10
CTM8 F	GGACTATGTATACAGCTGGAT	0.12
CTM8 R	ACCACCGCCAGTTAATAT	0.12
CTM12 F	CTGCTGACAAACTTGAAGAA	0.18
CTM12 R	TCTTGGTGATCCAAATGAAATT	0.18
CTM17 F	TTCATGGATGGAAGCAGT	0.08
CTM17 R	GGCACTGGATATTTCAGATAT	0.08
